# Probing Leader Cells in Endothelial Collective Migration by Plasma Lithography Geometric Confinement

**DOI:** 10.1038/srep22707

**Published:** 2016-03-03

**Authors:** Yongliang Yang, Nima Jamilpour, Baoyin Yao, Zachary S. Dean, Reza Riahi, Pak Kin Wong

**Affiliations:** 1 Department of Aerospace and Mechanical Engineering, The University of Arizona, Tucson, AZ 85721-0119, USA; 2 Institute of Opto-electronics Technology, School of Instrumentation Science and Opto-electronics Engineering, Beihang University, Beijing, 100191, P. R. China; 3 Biomedical Engineering Graduate Interdisciplinary Program, The University of Arizona, Tucson, AZ 85721-0119, USA; 4 Departments of Biomedical Engineering, Mechanical Engineering, and Surgery, The Pennsylvania State University, University Park, PA 16802, USA

## Abstract

When blood vessels are injured, leader cells emerge in the endothelium to heal the wound and restore the vasculature integrity. The characteristics of leader cells during endothelial collective migration under diverse physiological conditions, however, are poorly understood. Here we investigate the regulation and function of endothelial leader cells by plasma lithography geometric confinement generated. Endothelial leader cells display an aggressive phenotype, connect to follower cells via peripheral actin cables and discontinuous adherens junctions, and lead migrating clusters near the leading edge. Time-lapse microscopy, immunostaining, and particle image velocimetry reveal that the density of leader cells and the speed of migrating clusters are tightly regulated in a wide range of geometric patterns. By challenging the cells with converging, diverging and competing patterns, we show that the density of leader cells correlates with the size and coherence of the migrating clusters. Collectively, our data provide evidence that leader cells control endothelial collective migration by regualting the migrating clusters.

The endothelium forms the inner lining of blood vessels and plays essential roles in vascular biology^1^. It provides a functional barrier for retaining circulating blood, regulating blood-tissue exchange, recruiting blood cells, and controlling vascular tone. The integrity of the endothelium plays an important role in its physiological function. Upon mechanical injury, e.g., iatrogenic operations, endothelial cells migrate to heal the wound autonomously and maintain the functions of the vasculature. Endothelial cells crawl collectively atop the wound bed to restore the integrity of the endothelium. The migration of endothelial cells is modularly controlled to regulate cell motility, directed migration, cell-cell coordination, and cell density^2^,^3^. Calcium signaling components, such as phospholipase C, stromal interaction molecule 1 and diacylglycerol, are polarized at the leading edge to promote persistent forward migration^4^. Previous studies of collective cell migration, however, were performed primarily using epithelial cells. A better understanding of endothelial collective migration is essential for the development of novel therapeutics and tissue engineering approaches to treat endothelial dysfunction and vascular diseases.

The formation of leader cells are observed during epithelial wound closure^5^,^6^. In particular, leader cells with an aggressive phenotype emerge near the wound boundary and mechanically interact with follower cells to form multicellular migrating clusters^7^. Mechanical force and the Rho signaling pathway are known to modulate the formation of leader cells^8^,^9^,^10^,^11^. Inhibition of Rho signaling with Y-27632 increases the leader cell density at the leading edge. Recently, Notch1-Dll4 lateral inhibition is also shown to regulate leader cell formation during epithelial collective migration^12^. Despite its importance in vascular biology, the regulation of endothelial leader cells under various physiological conditions, such as different wound sizes and shapes, remain poorly understood. More importantly, the functional relationship between leader cells and follower cells in the migrating clusters has not been explored due to the challenge of adjusting the leader cell density systematically. The ability to non-invasively modulate the leader cell density will be invaluable for deciphering the functions of leader cells.

In this study, we develop a plasma lithography modulated would healing assay to study the regulation and function of leader cells. Collective migration of human umbilical vein endothelial cells (HUVEC), which is an established model system for human endothelial cells, is studied on geometric patterns created by surface plasma treatment^13^,^14^,^15^,^16^,^17^. In the assay, the geometry of the cell monolayer is controlled by spatially patterning the hydrophobicity of the substrate and collective migration is induced by removing a physical blocker to create a cell free region in the pattern^18^. The plasma lithography technique has been previously demonstrated for investigating several biological systems^19^,^20^,^21^. The formation of leader cells and the migration rate of the monolayer are investigated in rectangular patterns of various dimensions. Converging, diverging and competing patterns are also designed to perturb the leader cell density non-invasively. The leader cells and follower cells in the migrating cluster are characterized by immunostaining, time-lapse microscopy and particle image velocimetry (PIV). The influences of the leader cell density on the migrating cluster and the overall migration rate of the monolayer are investigated to elucidate the functions of leader cells.

## Results

### The density of migrating clusters is independent of the pattern width

**Figure 1:**
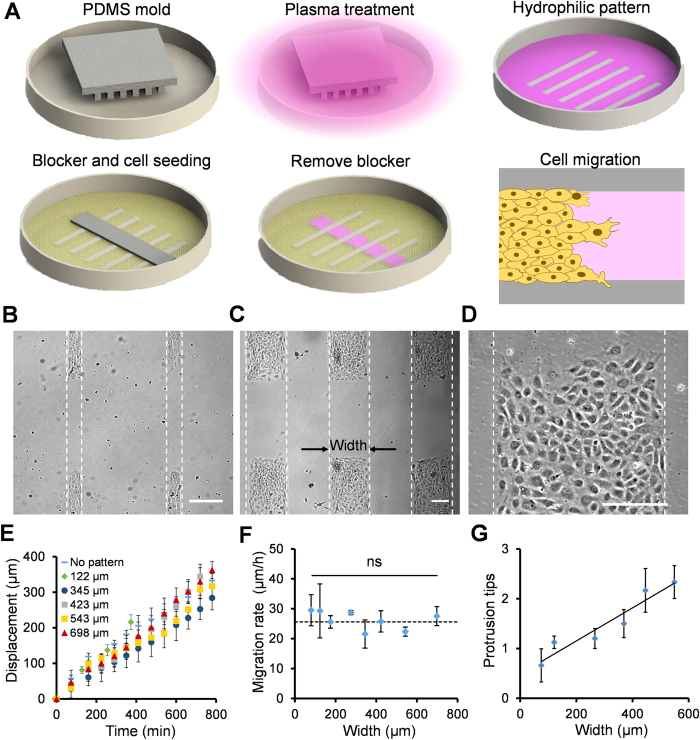
Endothelial collective migration in geometrically confined microenvironments. (**A**) Schematic of the plasma lithography modulated wound healing assay. In this assay, hydrophilic cell adhesion patterns are created by physical shielding of plasma treatment with a PDMS mold. A PDMS blocker is placed in the plasma lithography patterned polystyrene dish before cell seeding. After a cell monolayer is formed, the PDMS blocker is removed to create a cell free region for studying collective migration. (**B**–**D**) Representative images of rectangular cell patterns with different widths. Scale bars, 200 μm. (**E**) The displacement of cells in patterns with different widths. (**F**) The migration rate of cells in patterns with different widths. The dotted line indicates the migration rate without plasma lithography patterning. Data represent mean ± s.e.m. (ns: not significant, ANOVA). (**G**) The average number of protrusion tips for cells in patterns with different widths. The solid line represents the best fit line. Data represent mean ± s.e.m.

A plasma lithography modulated wound healing assay was developed to study endothelial collective migration (Fig. 1A and supplementary Fig. S1A). In this assay, a polydimethylsiloxane (PDMS) mold was applied to shield specific regions of a polystyrene substrate from air plasma treatment. Selective plasma treatment created hydrophilic patterns that promote cell adhesion. A cell free region for cell migration was generated by placing a PDMS blocker onto the appropriate location of the polystyrene dish before cell seeding^18^. Upon the release of the blocker, the cell monolayer migrated toward the cell free region. Consistent with other injury-free assays^22^,^23^,^24^, the release of contact inhibition was sufficient to induce collective migration on the patterns (supplementary Fig. S1B). The technique allowed us to control the width, gap size, and length of the pattern independently and to study collective migration in patterns with the dimension from a single cell to a monolayer (Fig. 1B–D and supplementary Fig. S1C). In our experiments, the migration rate of HUVECs was similar between the PDMS blocker assay and the scratch assay with and without plasma lithography geometric confinement (supplementary Fig. S1B). These data support the applicability of the plasma lithography modulated wound healing assay for probing endothelial collective migration.

We investigated endothelial collective migration by designing rectangular patterns with different dimensions systematically (Fig. 1B–D). The cells migrated at a constant speed upon the removal of the PDMS blocker in all patterns (Fig. 1E). The migration rate was approximately constant, ~30 μm/h, and was independent of the pattern width from 75 μm to the entire monolayer (Fig. 1F). The migration rate was also independent of the initial pattern length and gap size (supplementary Fig. S2). We examined the leading edges of the migrating endothelia. The leading edge evolved from a straight boundary to an irregular leading edge with the formation of protrusion tips and migrating clusters (supplementary Fig. S3). The migrating clusters are multicellular structures with coherent velocities (both migration rate and direction) near the leading edge. The formation of multicellular migrating clusters was also reported in collective migration of epithelial cells^25^,^26^,^27^. Our results indicated the average number of protrusion tips and migrating clusters increased linearly with the pattern width (Fig. 1G). The densities of protrusion tips and migrating clusters were approximately constant (~250 μm per cluster) for patterns of various widths.

### Leader cells and migrating clusters emerge at the leading edge

**Figure 2:**
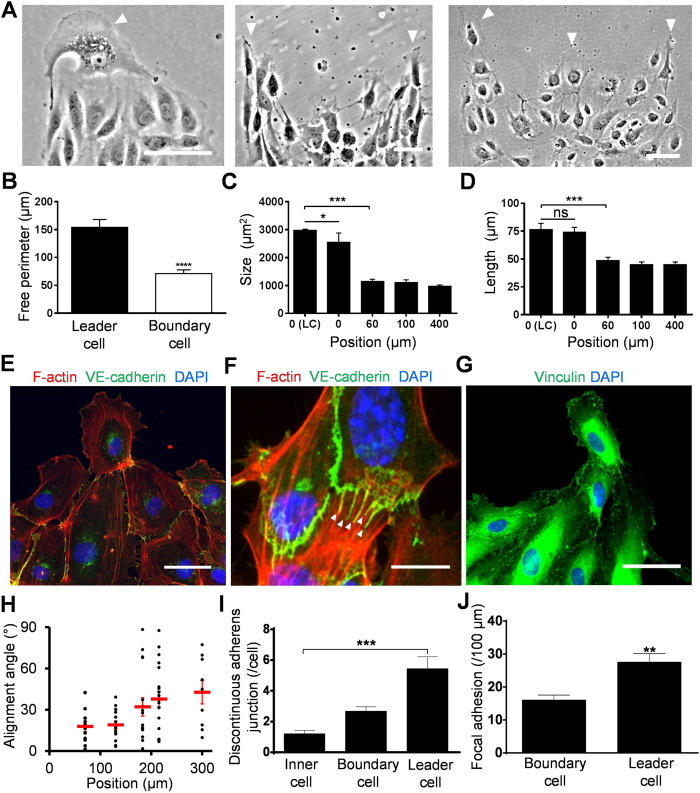
Characteristics of leader cells and follower cells in migrating clusters. (**A**) Representative images of leader cells in rectangular patterns with different widths. White arrow heads indicate the position of leader cells. Scale bars, 100 μm. (**B**–**D**) The free perimeter (**B**), size (**C**), and length (**D**) of leader cells compared to other cells at the boundary and in the inner region of the monolayer. (LC, leader cell; n = 7 for leader cells; n = 13 for boundary cells; n = 10 for cells in the monolayer; ns: not significant, * P< 0.05, **P < 0.01, ***P < 0.001). (**E**,**F**) Representative immunofluorescence images of F-actin (red), VE-cadherin (green), and nuclei (blue) in leader cells and follower cells. White arrow heads indicate the discontinuous adherens junctions. Scale bars, 50 μm. (**G**) Representative immunofluorescence image of vinculin (green) and nuclei (blue) in leader cells and follower cells. Scale bar, 50 μm. (**H**) The alignment angle of follower cells relative to the leader cells. The cell alignment angle was defined by the orientation of the actin stress fibers. (**I**) The number of discontinuous adherens junctions per cells in leader cells, cells at the boundary, and cells in the monolayer. (**J**) The number of observable focal adhesions in leader cells and other cells at the boundary. Data represent mean ± s.e.m. (n = 3 for leader cells and n = 7 for boundary cells; *P < 0.05, **P < 0.01, ***P < 0.001). Experiments were repeated at least three times independently.

We further characterized the protrusion tips and migrating clusters during endothelial collective migration. Examination of the leading edge revealed the formation of endothelial leader cells with an aggressive phenotype at the protrusion tip (Fig. 2A). These leader cells were morphologically distinctive compared to other cells at the leading edge and in the cell monolayer. Importantly, the leader cells developed active lamellipodia and the free perimeter of the leader cells was twofold larger than that of other cells at the boundary (Fig. 2B). The size of leader cells was also enhanced compared to cells in the monolayer (Fig. 2C). Most endothelial cells at the boundary displayed a polarized, spindle-like morphology (Fig. 2D). The length of leader cells (along the longest axis) was significantly longer than the cells in the inner region of the monolayer. Based on these observations, cells with a large free perimeter due to lamellipodia formation were applied to quantitatively identify leader cells in this study. We further characterized the leader cells and cells in the migrating clusters. Immunostaining revealed that leader cells developed prominent stress fibers (Fig. 2E), connected follower cells via peripheral actin cables and adherens junctions (Fig. 2F), and displayed enlarged vinculin-based focal adhesions (Fig. 2G). Discontinuous adherens junctions, which are perpendicular to the cellular boundaries^28^, were observed between leader cells and the adjacent follower cells (Fig. 2F and supplementary Fig. S4).

The alignment of the follower cells relative to the leader cells was analyzed in the migrating clusters (Fig. 2H and supplementary Fig. S4A–C). The cell orientation was defined by the actin stress fibers, which generally aligned along the longest axis of the cells. The mean alignment angle increased gradually from less than 10° in the adjacent follower cells and reached ~45° (i.e., random alignment) in approximately 250 μm. This length scale is compatible to the density of the protrusion tip. The formation of discontinuous adherens junctions, which link to the actin cytoskeleton and associate with force-dependent remodeling of endothelial cells, was analyzed (Fig. 2F and supplementary Fig. S4D–G). The number of observable discontinuous adherens junctions in leader cells was at least twofold higher than that of other cells at the leading edge, and fourfold higher than cells in the inner region (Fig. 2I). Furthermore, leader cells had a high number of enlarged focal adhesions compared with other cells at the boundary (Fig. 2J). These results suggest endothelial leader cells mechanically interact with follower cells to form migrating clusters.

### Velocity mapping of migrating clusters in endothelial collective migration

**Figure 3:**
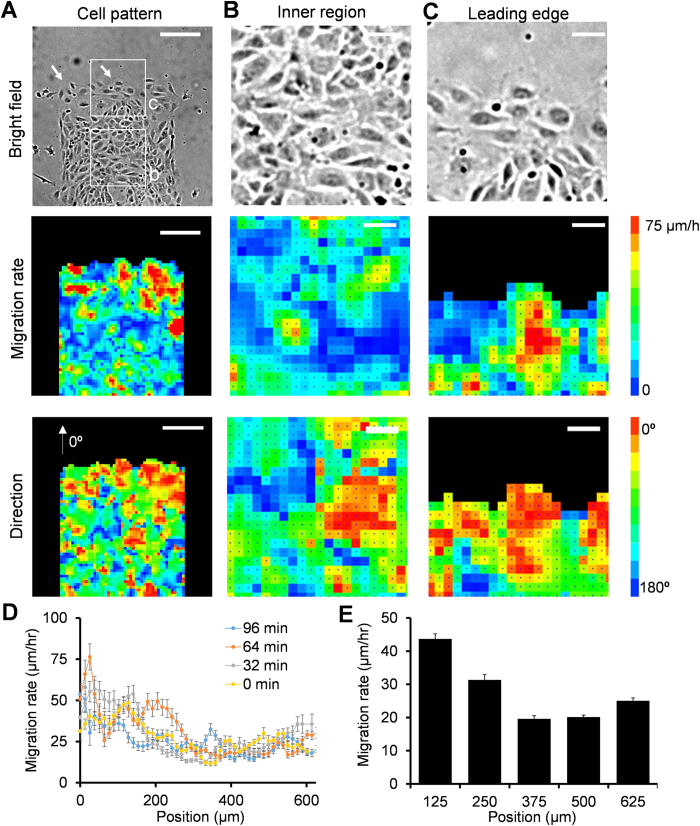
Velocity mapping reveals the formation of migrating clusters with coherent velocities. (**A**–**C**) Representative images (top) and PIV analysis (middle and bottom) of cell migration in a rectangular pattern. White arrow heads indicate the locations of leader cells. White squares indicate the zoom-in regions in (**B**,**C**). Scale bars, (**A**) 200 μm and (**B**,**C**) 50 μm. (**D**) Instantaneous velocity distribution at different time points. (**E**) Average velocity distribution at different positions from the leading edge. Data represent mean ± s.e.m of over 100 time points. Experiments were repeated three times independently.

The morphology of the leading edge and the alignment of follower cells suggest the formation of migrating clusters. The spatiotemporal velocity distribution was quantified using a PIV algorithm to characterize the migratory behaviors of the migrating clusters (Fig. 3A–D). A large fluctuation of the migration rate and direction was observed in the leader cells and the migrating clusters (supplementary video S1). On average, the migration rate was maximized at the leading edge and decreased gradually from the leading edge to the inner region of the cell monolayer (Fig. 3E). The average migration rate distribution was in good agreement with previous studies of collective migration^24^,^29^. The instantaneous velocity distribution suggested the formation of migrating clusters with coherent migration rate and direction near the leading edge (Fig. 3A–C). Each cluster was generally associated with a leader cell and the length scale of the migrating cluster was ~250 μm. The length scale of migrating clusters was estimated by inspecting cells with similar velocities (supplementary video S1). The length scale of the migrating cluster could also be determined quantitatively by autocorrelation of the velocity distribution or analyzing cells with migration rate over 30 μm/h (supplementary Fig. S5). The length scale of the migrating cluster was consistent with the density of leader cells and the region of follower cells aligned toward the leader cells, supporting the notion that leader cells interact with follower cells to form migrating clusters.

### Leader cell density correlates with the size and coherence of migrating clusters

**Figure 4:**
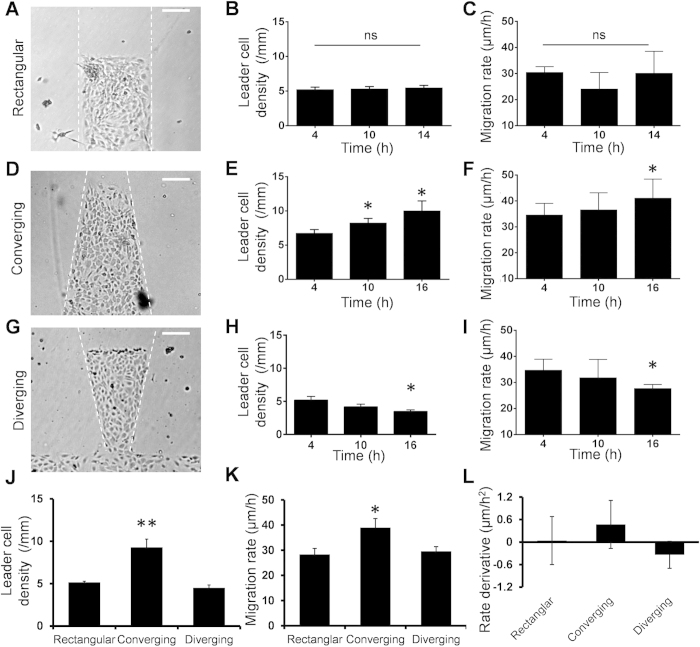
Converging and diverging patterns modulate leader cell density and migration rate. (**A**–**C**) Cell migration in rectangular patterns with a constant width. (D-F) Cell migration in converging patterns. (**G**–**I**) Cell migration in diverging patterns. Phase contrast images of the cell pattern initially, the density of leader cell, and the migration rate of cells at different time points were shown for different patterns. (**J**,**K**) Comparison of the leader cell density and migration rate. (**L**) The rate of change of the migration rate (rate derivative) for cells in rectangular, converging, and diverging patterns. Data represent mean ± s.e.m. (n = 4; ns: not significant, *p < 0.05, **p < 0.01).

To elucidate the roles of leader cells in the regulation of migrating clusters, we designed diverging and converging patterns for perturbing the leader cell density (supplementary Fig. S6). In particular, we seeded endothelial cells into trapezoidal patterns with the width of the leading edge that is either constant (rectangular), increasing (diverging) or decreasing (converging). For the rectangular pattern, the density of leader cells and the migration rate were constant in the duration of the experiment (Fig. 4A–C). In the converging pattern, leader cells with active lamellipodia were formed with a density similar to the rectangular pattern initially (Fig. 4D and supplementary video S2). As the cells migrated, the width of leading edge decreased while the number of leader cells appeared to be constant. Consequently, the leader cell density increased continuously and a high density of leader cells was observed at the leading edge (Fig. 4E). The migration rate of the cell monolayer also increased continuously and correlated with the leader cell density (Fig. 4F). In the diverging pattern, the leading edge widened as the cell clusters migrating forward (Fig. 4G). As a result, the leader cell density in the diverging pattern decreased (Fig. 4H) and the migration rate also showed a decreasing trend in the duration of the experiment (Fig. 4I).

We compared the leader cell densities and overall migration rates of different patterns. Remarkably, the migration rate correlated with the leader cell density (Fig. 4J,K). To exclude the uncertainty due to the area variation in the converging and diverging patterns, we also calculated the effective displacement and migration rate based on the area increments (Supplementary Fig. S7). In agreement, the effective migration rate increased in the converging pattern and decreased in the diverging pattern, which correlated with the leader cell density. The rate of change of leader cell density also correlated with the rate of change of migration rate (Fig. 4L).

**Figure 5:**
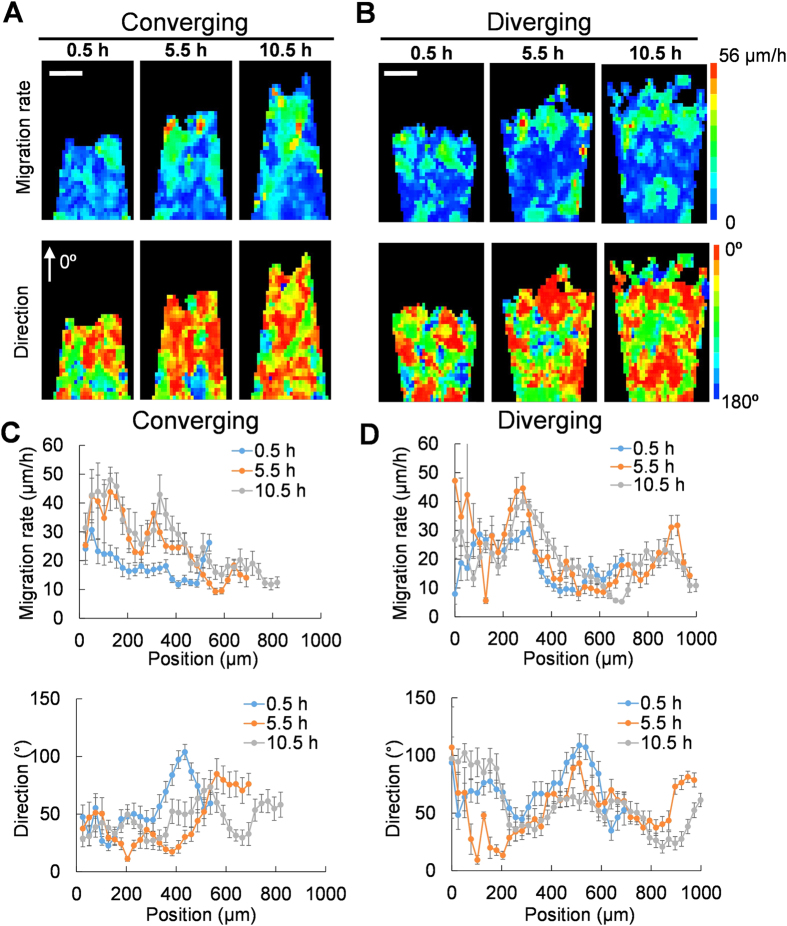
Geometric patterns modulate the size and coherence of migrating clusters. (**A**,**B**) The spatiotemporal distributions of the velocity for cells on converging and diverging patterns. (**C**,**D**) The position dependence of the instantaneous migration rate in converging and diverging patterns. The position dependence of the migration direction in converging and diverging patterns. Data are representative of five independent experiments.

We further investigated the functions of leader cells by analyzing the spatiotemporal velocity distribution in diverging and converging patterns (Fig. 5A,B). For all patterns, the average migration rate was maximized at the leading edge and decreased gradually in the inner region of the pattern. The maximum instantaneous migration rates were similar in all patterns, indicating that the mobility of individual cells was not modified by geometric confinement. Formation of migrating clusters with coherent velocity was observed in both converging and diverging patterns (Fig. 5A,B and supplementary Fig. S8A). In the converging pattern, the size of the cell cluster was ~250 μm initially (Fig. 5A). As the leader cell density increased, the size of the migrating cluster also appeared to increase (Fig. 5C and supplementary Fig. S8A and Video S2). Remarkably, the size of migrating clusters with coherent velocity was over 400 μm. In contrast, the migrating cluster in the diverging pattern was smaller and the average size of the migrating cluster with coherent velocity decreased to less than 200 μm (Fig. 5B). Examination of the migration direction also revealed a wide distribution of migration directions in the diverging pattern (Fig. 5D and supplementary Fig. 8B and Video S3). As indicated by the migration direction, the cells in converging patterns migrated directionally toward the cell free region compared to cells in the diverging pattern. The coherence of the migrating cluster provides an explanation to the difference in the overall migration rate despite the maximum instantaneous migration rates were similar in all patterns.

### Leader cells control migrating clusters in competing cell structures

**Figure 6:**
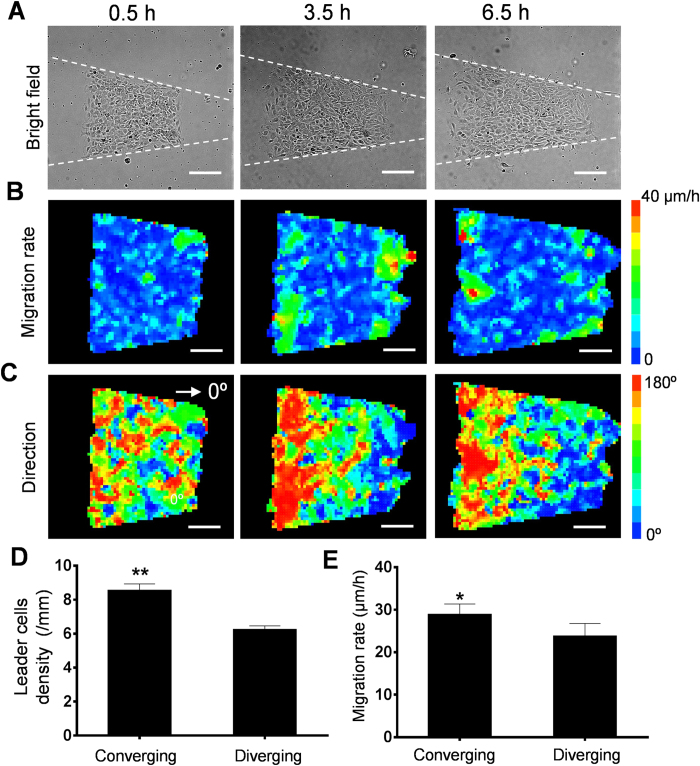
The size and coherence of the migrating clusters in competing cell patterns. (**A**) Representative images of the competing wound healing assay at 0.5 hours, 3.5 hours, and 6.5 hours after removal of the PDMS blocker. (**B**,**C**) The migration rate and migration direction of the competing cell structure. (**D**,**E**) The density of leader cells and the migration rate at the diverging and converging ends 6.5 hours after removal of the PDMS blockers. Images are representative of five independent experiments. Data represent mean ± s.e.m. (n = 4; *p < 0.05, **p < 0.01).

To further investigate the effects of leader cells on the migrating clusters, a competing cell structure was created by placing PDMS blockers at both ends of the pattern before cell seeding (Fig. 6 and supplementary video S4). For competing cell structures on rectangular patterns, the migration rates were similar in both ends. The migration rate of the competing cell structure was comparable with the migration rates in dead end and open end patterns (supplementary Fig. S9). For trapezoidal patterns, the cell migration rates at both ends were observed and the velocity distributions were analyzed using PIV (Fig. 6A–C and supplementary Video 4). The initial length of the competing cell monolayer was 600–800 μm. Leader cell formation and collective migration toward the cell free regions were observed at both ends. Initially (<0.5 h), small migrating clusters were developed near the boundaries towards the corresponding cell free regions. The migration direction was random in the inner region of the cell pattern. As the cells migrated, the size of the coherent migrating cell cluster increased. After three hours, the leader cell density and the size of the migrating cell cluster were significantly larger in the converging end compared to the diverging end (Fig. 6B–E). The size of the cell cluster moving toward the converging end was ~400 μm while the value was ~200 μm for the diverging end. The size and coherence of the cell clusters migrating in each direction were robustly determined by categorizing cells into left and right, considering the histogram of the migration direction, and analyzing the speed distribution (supplementary Fig. S10). These data further support that notion that the leader cells regulate the size and coherence of the migrating cell cluster to control the overall migration rate.

## Discussion

In this study, we investigate the regulation and function of leader cells during endothelial collective migration. Pervious investigations of leader cells were primarily performed using epithelial cells. Our study represents one of the first comprehensive investigation of endothelial leader cells. Endothelial leader cells locate at the protrusion tip and display an aggressive phenotype with prominent stress fibers, ruffling lamellipodia and enlarged focal adhesions. The stress fibers are known to physically link endothelial cells together via VE-cadherin based adherens junctions to transmit mechanical force^30^,^31^. Immunostaining also reveals the formation of peripheral actin cables and discontinuous adherens junctions, which indicate mechanical coupling between leader and follower cells in the migrating cluster. The formation of migrating clusters with coherent velocities was identified by time-lapse microscopy and PIV analysis. These results collectively suggest that endothelial leader cells emerge and mechanically interact with follower cells to form migrating clusters during endothelial collective migration.

The plasma lithography modulated wound healing assay allows us to perturb the density of leader cells and study its functions on the overall migration process. Within the duration of the experiment (16 hours), the numbers of leader cells in the patterns were not changed significantly. As the cells migrate in the converging pattern, the leader cell density increases effectively in the narrower region. In contrast, the leader cell density decreases in the diverging pattern. This ability to modulate the leader cell density represents a unique, physical approach to study the function of leader cells. The technique does not require drug treatment (e.g., Rho kinase inhibitor and Notch inducer/inhibitors) or genetic modification^8^,^9^,^10^,^11^,^12^, which may perturb multiple signaling pathways and cell phenotypes. Unlike laser ablation and micromanipulation^11^,^12^, geometrical confinement is non-invasive and does not require heating or mechanical injury, which may affect the cell behaviors. The plasma lithography modulated wound healing assay provides a useful tool to study the function of leader cells.

An interesting observation in this study is that the size and coherence of migrating clusters correlate with the density of leader cells. Several lines of evidence support that the migrating clusters are regulated by leader cells. Firstly, the correlation between the leader cell density and the migrating cluster was consistently observed in rectangular, converging, diverging and competing patterns, which systematically adjust the leader cell density. Secondly, the length scale of the migrating clusters with coherent velocity was in quantitative agreement with the density of leader cells and the region of follower cells aligned toward the leader cells. Thirdly, the migrating clusters were similar in different patterns initially. The changes in the migrating clusters were only observed several hours after the removal of the PDMS blocker. The changes in the migrating cluster correlate with the time scale of the increase or decrease of the leader cell density. Furthermore, the mobility of individual cells and migrating clusters was similar with and without patterning, confirming that the influence of leader cell density was not triggered by the geometric boundary. Lastly, the results were observed consistently with different initial widths in the trapezoidal pattern by controlling the position of the PDMS blocker. Along with immunostaining and PIV analyses, these results suggest that endothelial leader cells modulate collective migration by regulating the migrating clusters. The leader cells may also regulate collective migration by modulating other characteristics of the migrating clusters, such as the proliferation rate.

In summary, we demonstrate a plasma lithography modulated wound healing assay for investigating collective cell migration. The technique enables a novel approach to study the regulation and functions of leader cells. By perturbing the leader cell density, our results provide evidence that the density of leader cells correlates with the size and coherence of the migrating clusters, which may represent a physiological mechanism to control endothelial collective migration for diverse wound geometries. Future investigation should be performed to further investigate the functions of leader cells and to elucidate the regulation of migrating clusters.

## Method and Materials

### Cell culture

Human Umbilical Vein Endothelial Cells (BD Biosciences) from passage 2–8 were used in the experiment. The cells were cultured in Medium 200 with low serum growth supplement (Life Technologies) and 0.1% gentamicin (GIBCO) at 37 °C with 5% CO_2_. The medium was renewed every two days.

### Wound healing assay

To perform the blocker assay, a thin slab of PDMS was prepared in a petri dish at a 10:1 base to curing agent ratio. The PDMS slab was cured on a hot plate for 4 hours at 65 °C. PDMS blockers were cut to the appropriate size using a razor blade sterilized with 70% ethanol. The PDMS blockers were placed in tissue culture dishes or 24 well plates before cell seeding. A conformal contact was created between the substrate and the PDMS blocker. After forming the cell monolayer, the PDMS blocker was carefully removed to create a cell free region for cell migration.

### Plasma lithography modulated wound healing assay

Plasma lithography was preformed to create geometric patterns for cell adhesion and migration^14^,^15^,^16^,^17^. Briefly, a master mold was produced by patterning SU8 on a silicon substrate. PDMS base and curing agent were mixed at 10:1 ratio and poured on the master. The PDMS sample was incubated for 4 hours at 65 °C to create the PDMS mold. The mold was placed onto the polystyrene petri dish with conformal contact and was exposed to air plasma at 1000 mTorr (Harrick). Shielding of the substrate by the PDMS mold from plasma treatment spatially functionalized the substrate to create patterns for cell adhesion. The PDMS blocker assay was performed on the plasma lithography defined patterns to study endothelial collective migration in geometric confinement.

### Immunofluorescence staining

Cells were stained for F-actin with Alexa Fluor® 555 tagged phalloidin (Life Technologies), for vinculin based focal adhesions with monoclonal anti-vinculin antibody (Sigma Aldrich), and for VE-cadherin based adherens junctions with VE-cadherin rabbit monoclonal antibody (Cell Signaling Technology). The nuclei were stained using ProLong® Gold antifade reagent with DAPI (Invitrogen). All antibodies were diluted using antibody dilution buffer (Ventana Medical Systems). Samples were fixed with 4% paraformaldehyde (Electron Microscopy Sciences, # 15714) for 10 minutes after rinsing twice with warm PBS, then permeabilized with 0.1% triton-x 100 (Sigma Aldrich) for 15 minutes. The sample was covered with a glass cover slip and sealed with nail polish. Images were captured using a Nikon TE-2000 epi-fluorescence microscope.

### Live cell imaging and particle image velocimetry

Live cell imaging for velocity mapping was performed using a microscope stage with 5% CO_2_ at 37 °C. The microscope stage was loaded onto a Nikon TE-2000 microscope. Time-lapse images were analyzed using JPIV to determine the spatiotemporal velocity distribution. The interrogation window was selected to be 12.5 μm, which is compatible to the size of a cell. To compare the migration direction of cells, the direction toward the cell free region was defined as 0°. The data were analyzed using MATLAB.

### Statistical analysis

Student’s *t*-tests were performed to compare between experimental groups. For comparing multiple groups, a one-way analysis of variance and Tukey’s post hoc test were used. Data represent mean ± s.e.m. Experiments were performed in triplicate and repeated at least three times independently. Statistically significant P values were assigned as follows: ns: not significant, *P < 0.05, **P < *0.01* or ***P < *0.001*.

## Additional Information

**How to cite this article**: Yang, Y. *et al.* Probing Leader Cells in Endothelial Collective Migration by Plasma Lithography Geometric Confinement. *Sci. Rep.* **6**, 22707; doi: 10.1038/srep22707 (2016).

## Supplementary Material

Supplementary Information

Supplementary Video 1

Supplementary Video 2

Supplementary Video 3

Supplementary Video 4
