# Supplementary material for: Probing Leader Cells in Endothelial Collective Migration by Plasma Lithography Geometric Confinement
Source: Sci Rep. 2016 Mar 3;6:22707. doi: 10.1038/srep22707 (PMC4776176; doi:10.1038/srep22707)
Supplement: Supplementary Information [file srep22707-s1.doc]

**Supplementary Information**

Probing Leader Cells in Endothelial Collective Migration

by Plasma Lithography Geometric Confinement

Yongliang Yang^a^, Nima Jamilpour^a^, Baoyin Yao^a,b^, Zachary S. Dean^c^, Reza Riahi,^a^ Pak Kin Wong^a,c,d*^

^a^Department of Aerospace and Mechanical Engineering, The University of Arizona, Tucson, AZ 85721-0119, USA

^b^Institute of Opto-electronics Technology, School of Instrumentation Science and Opto-electronics Engineering, Beihang University, Beijing, P. R. China, 100191

^c^Biomedical Engineering Graduate Interdisciplinary Program, The University of Arizona, Tucson, AZ 85721-0119, USA

^d^Departments of Biomedical Engineering, Mechanical Engineering, and Surgery, The Pennsylvania State University, University Park, PA 16802, USA

* Corresponding author: E-mail: pak@engr.psu.edu; Tel: +1-814-863-5267.


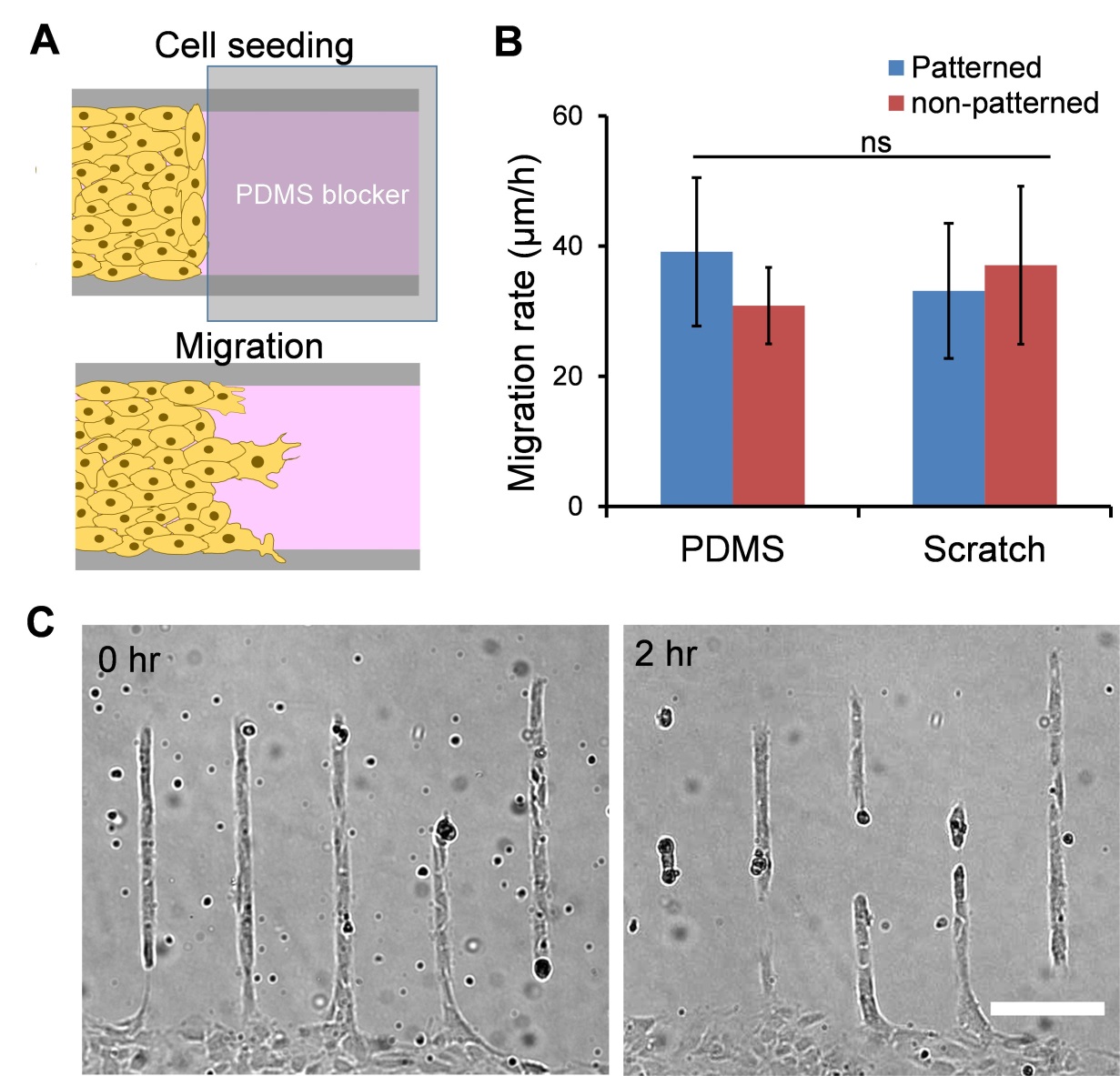


**Figure S1. Plasma lithography geometric confinement for probing endothelial collective migration.** (A) Schematic of the plasma lithography modulated wound healing assay. Endothelial cells were patterned on plasma treated hydrophilic areas (pink). Cell-free region for cell migration was created by removal of the PDMS blocker. (B) The migration rate was independent of the assay (PDMS or scratch) with and without plasma lithography patterning. The cells were seeded on 200 µm wide rectangular patterns. There is no statistically significant difference (ns: not significant, ANOVA). (C) Plasma lithography patterned HUVEC on rectangular patterns with the width of a single cell. Cell detachment was observed after 2 hours and data were not analyzed. The pattern width was 20 µm. Images are representative from three independent experiments. Scale bar, 100 µm.

**
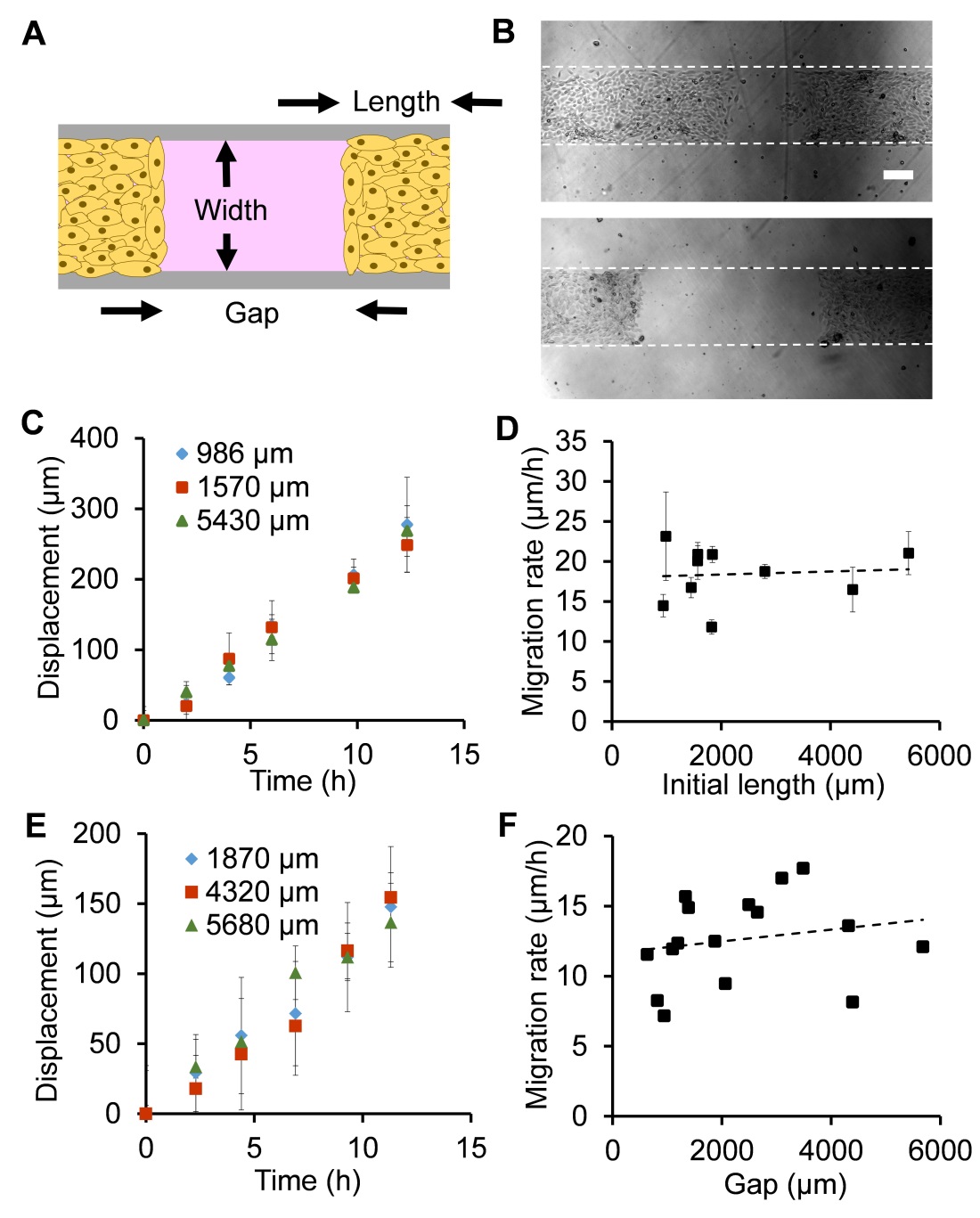
**

**Figure S2. The migration rate is independent of the length and gap of the cell pattern.** (A) Schematic illustration of the plasma lithography modulated wound healing assay for independent control of the width, gap and length of the cell pattern. (B) Representative images of rectangular patterns with different lengths. The initial gap distance and length were controlled by positioning PDMS blockers with different sizes. White dotted lines indicate the plasma treated regions for cell migration. Images are representative from three independent experiments. Scale bar, 200 µm. (C) The displacement of HUVEC with different initial lengths. (D) The migration rate for cells in rectangular patterns with different initial lengths. Dotted line represents the best fit line determined by linear regression. (R^2^=0.0066). (E) The displacement of HUVEC with different initial gap sizes. (F) The migration rate for cells in rectangular patterns with different initial gap sizes. Dotted line represents the best fit line determined by linear regression (R^2^=0.039).

**
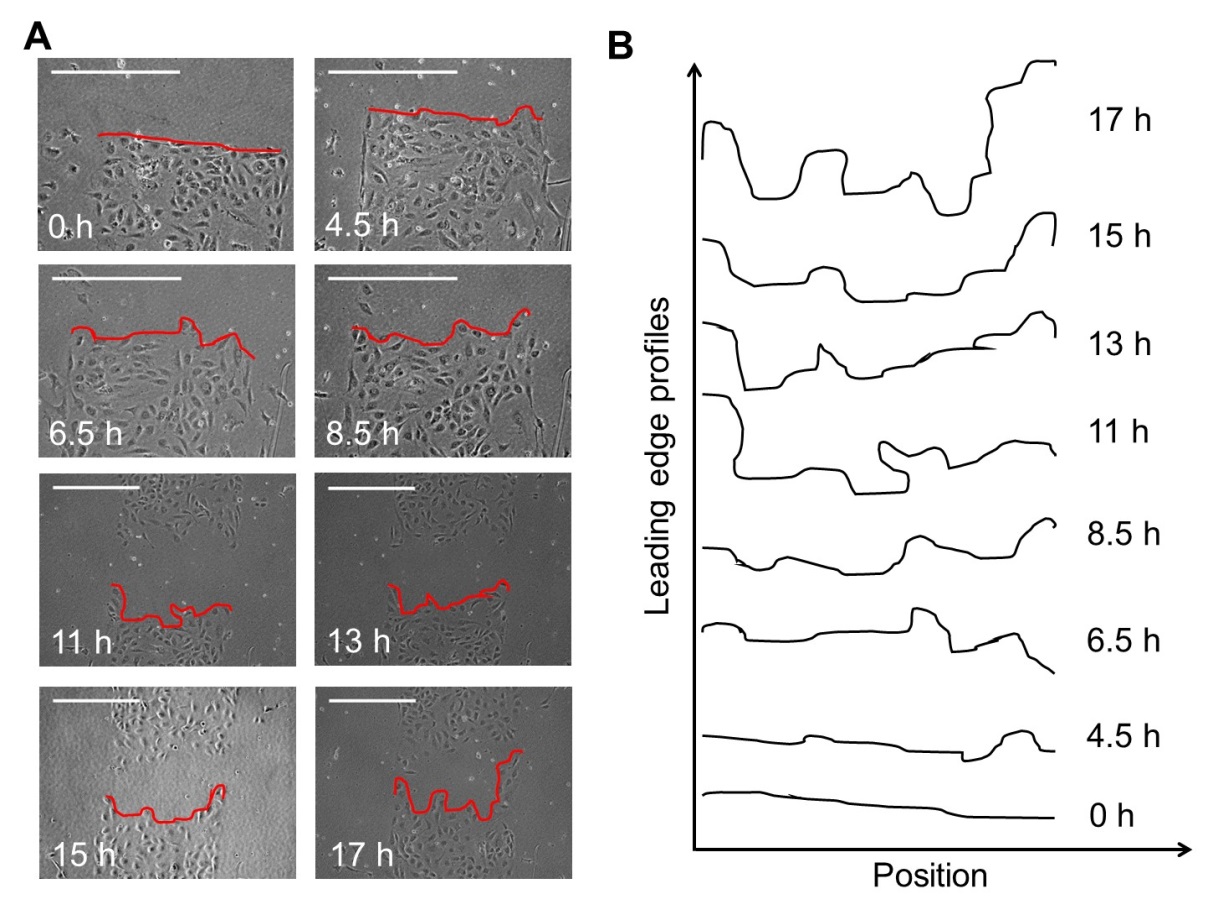
**

**Figure S3. Multicellular protrusion tips emerge at the leading edge.** (A) Representative images of the evolution of the wound boundary in a rectangular pattern. The width of the pattern was 700 µm. Scale bars, 500 µm. (B) The evolution of wound frontier during the wound healing process. The wound boundary profiles were shifted up for clarity.


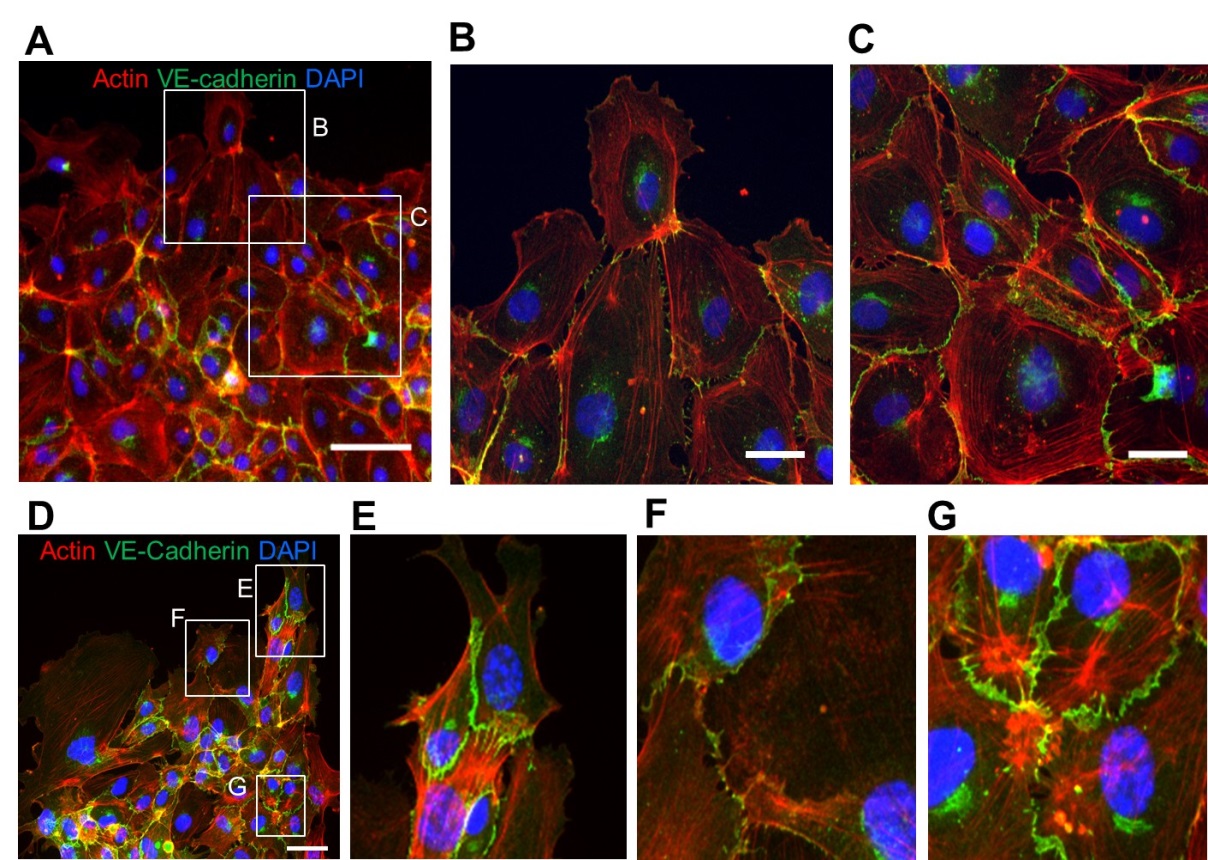


**Figure S4. Characterization of leader cells and follower cells during endothelial collective migration.** (A-G) Immunostaining of endothelial cells near the leading edge. HUVEC were stained for F-actin (red), VE-cadherin (green), and nuclei (blue). White squares indicate the zoom-in regions in B, C, E, F and G. Images are representative from three independent experiments. Scale bars, (A) 100 µm and (B-D) 50 µm.


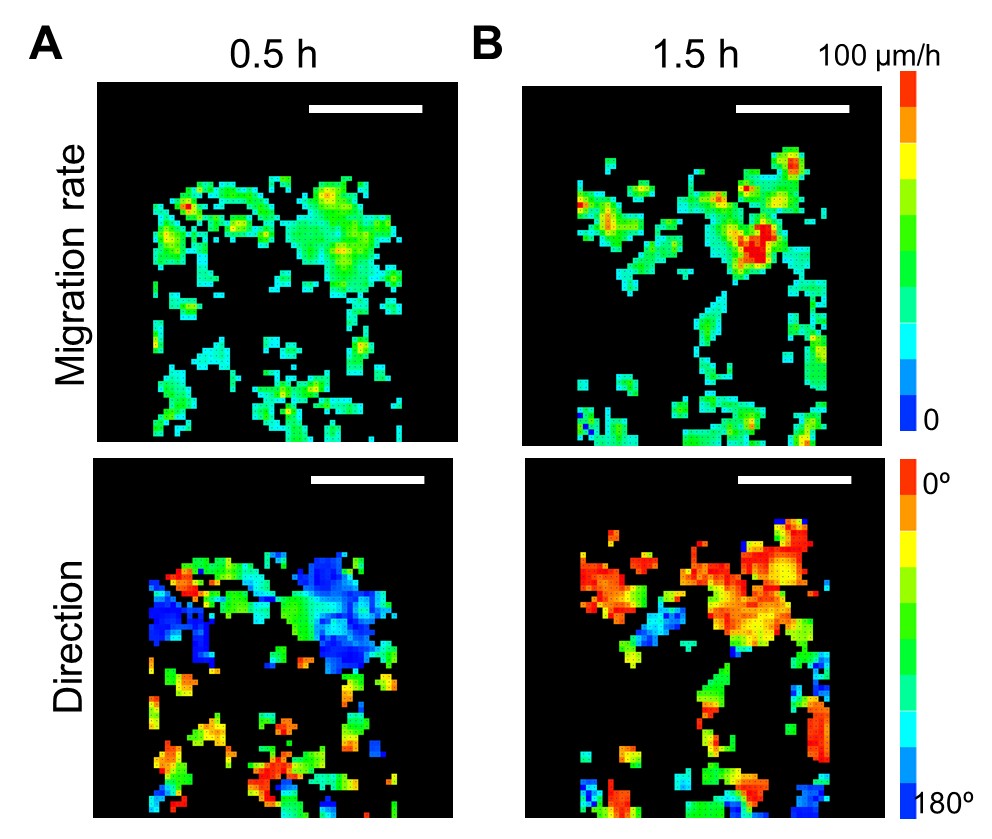


**Figure S5. Formation of migrating clusters in a rectangular pattern.** (A-B) Distribution of migration rate and direction in a rectangular pattern. Only cells with an instantaneous migration rate over 30 μm/h are shown to illustrate migration clusters with coherent migration rates and to determine the length scale of the migrating cluster. Scale bars, 200 μm.


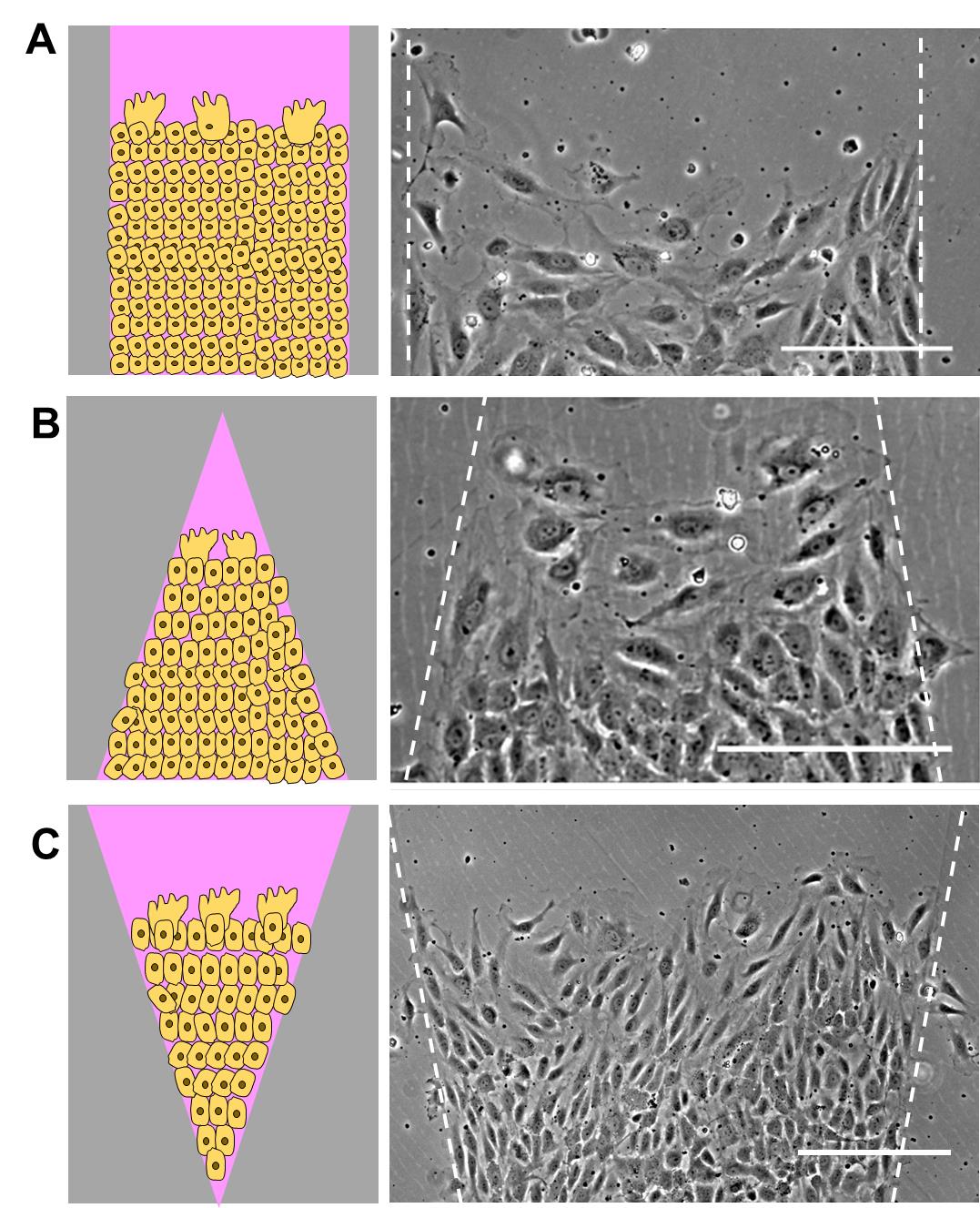


**Figure S6. Rectangular, converging and diverging cell patterns.** (A-C) Schematics and representative images of the (A) rectangular, (B) converging, and (C) diverging patterns. White dotted lines indicate the plasma treated regions for cell migration. Scale bars, 200 µm.

**
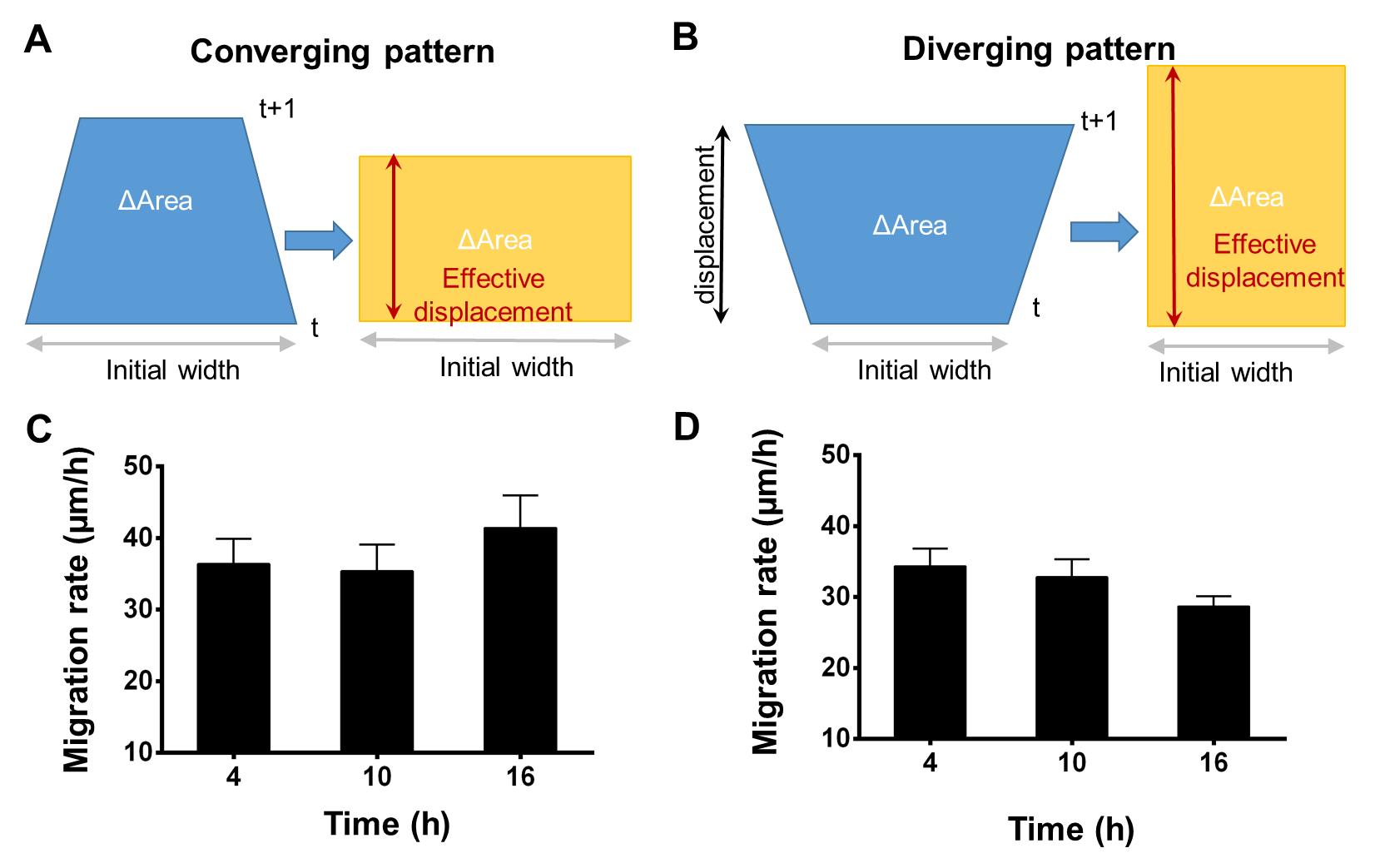
**

**Figure. S7. Effective displacement and migration rate in converging and diverging patterns.** (A-B) Schematic for calculating the effective displacement in converging and diverging patterns. (C-D) The migration rates of the converging and diverging patterns were calculated based on the equivalent displacement. The equivalent displacement is defined as the area increment at each observed time interval over the initial width of the leading edge.

**
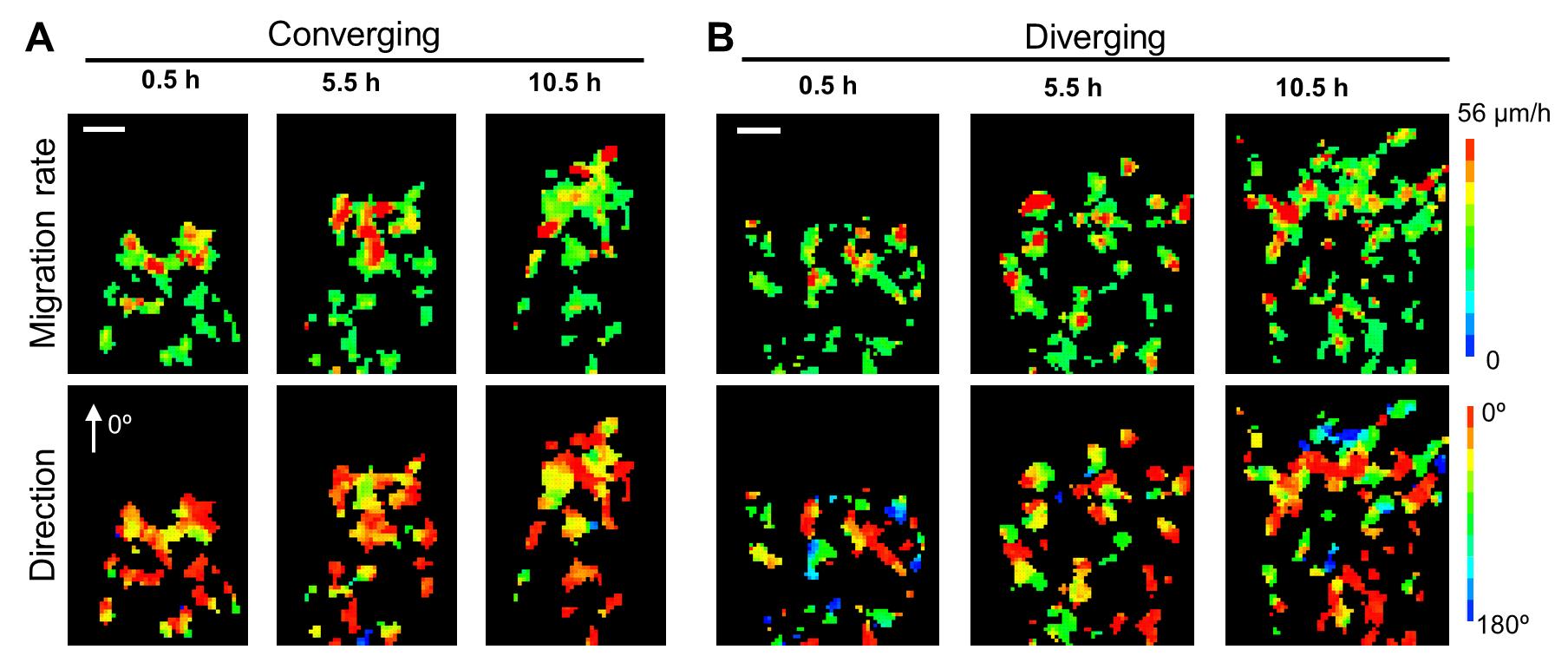
**

**Figure S8. Formation of migrating clusters in converging and diverging patterns.** (A-B) Distribution of migration rate and direction in converging and diverging patterns. Only cells with an instantaneous migration rate over 30 μm/h are shown to illustrate migration clusters with coherent migration rates and to determine the length scale of the migrating cluster. Scale bars, 200 μm.


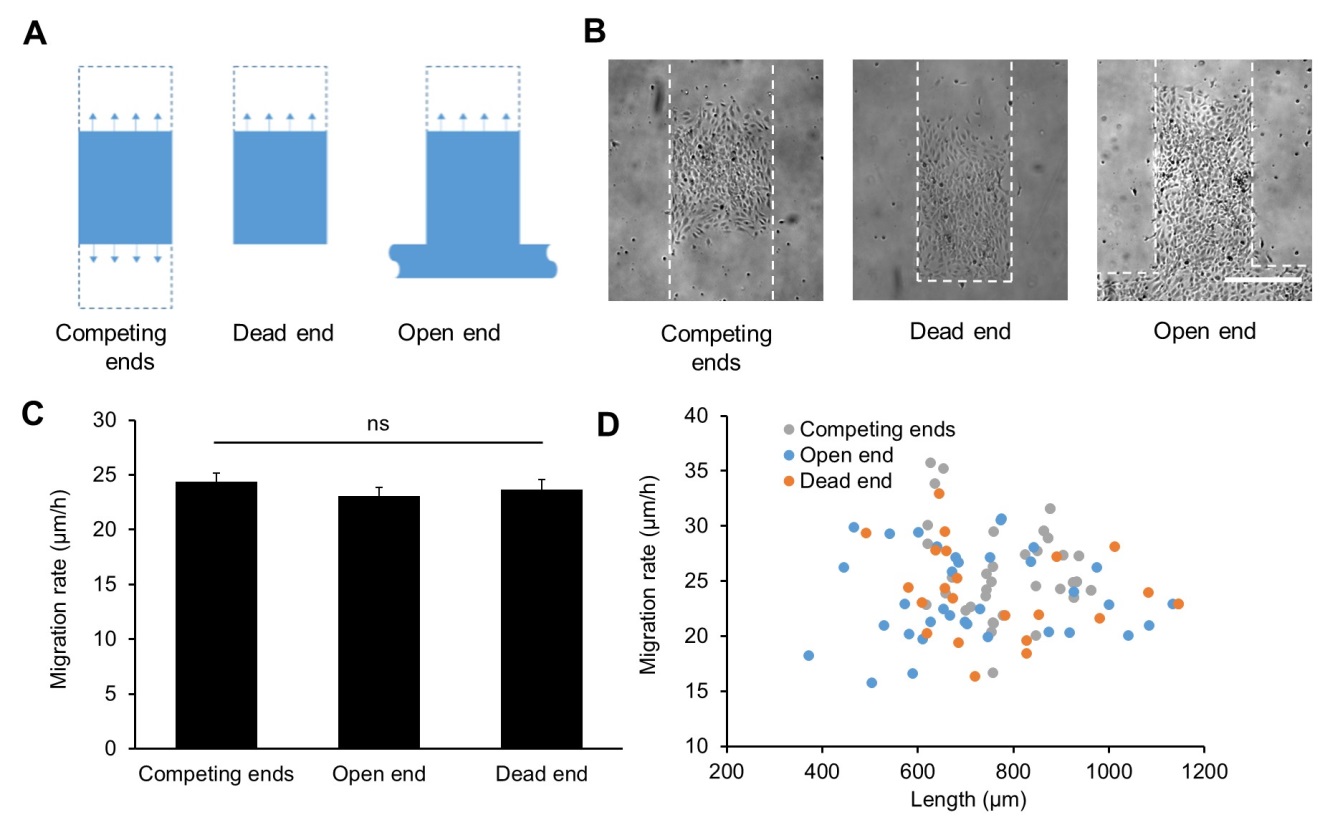


**Figure S9. Competing and dead end cell patterns.** (A) Three collective migration scenarios: competing ends, dead end, and open end. The cell patterns were created by modifying the plasma treated patterns and PDMS blocker positions. (B) Representative images of the three scenarios. White dotted lines indicate the plasma treated regions for cell migration. Scale bar, 200 μm. (C) Migration rates of cells patterns with competing ends, dead end, and open end (ns: not significant, ANOVA). (D) The effects of the initial length of the cell pattern on the migration rate.


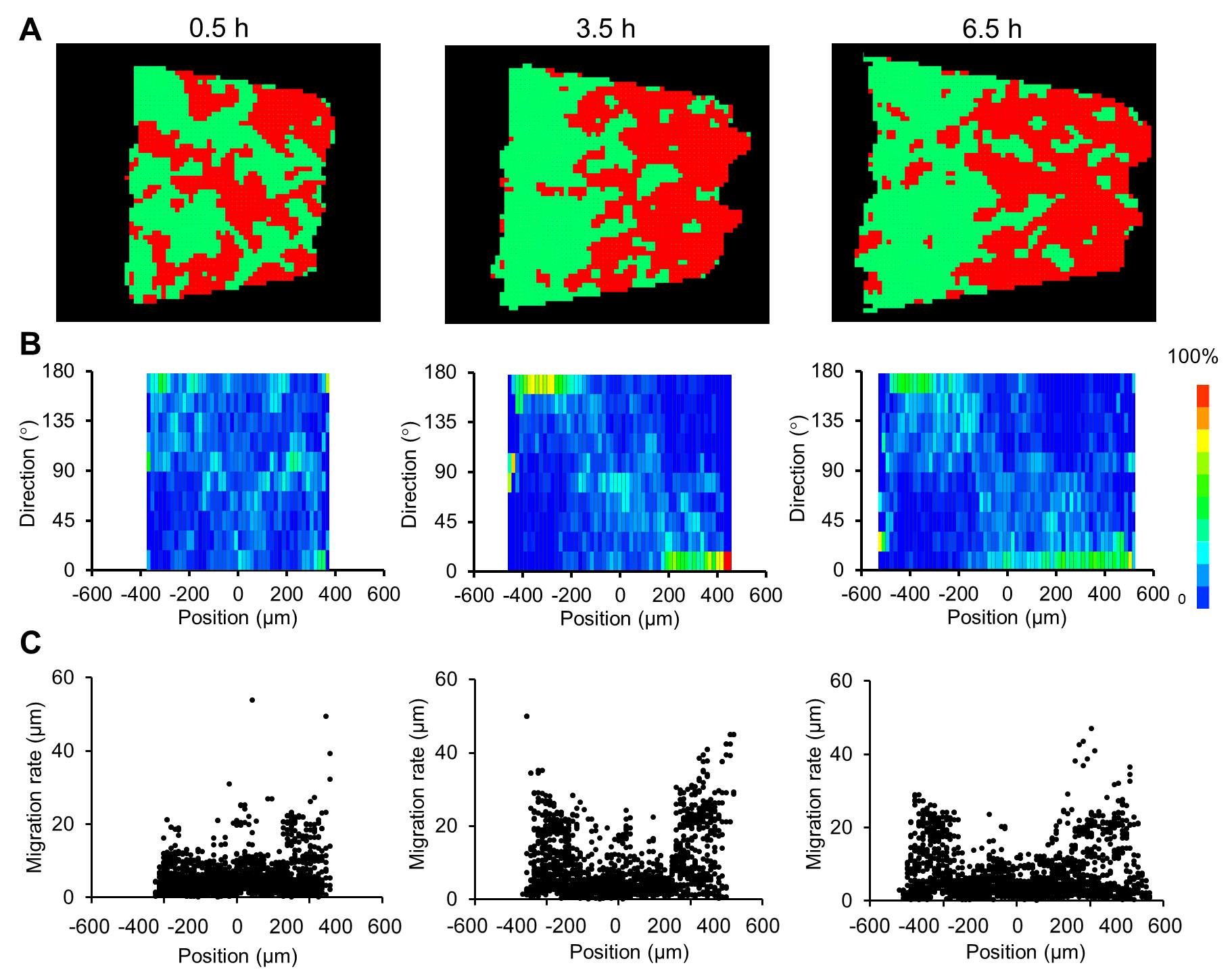


**Figure S10. Formation of migrating clusters in competing cell patterns.** (A) The migration direction of the cell pattern was mapped to illustrate the sizes of coherent migrating clusters in the competing pattern. Green and red colors indicate cells migrate towards left and right respectively. Scale bar, 200 μm. (B) The distributions of the migration angle are shown in the heat map illustrating the size of the coherent migrating clusters. (C) The spatial distributions of the migration rate in the competing pattern. Data are representative from three independent experiments.

**Supplementary Videos**

**Video 1: Endothelial collective migration on a rectangular pattern with PIV analysis**

HUVEC were seeded on a plasma lithography defined rectangular pattern created on a 60 mm petri dish. Cell migration was induced by the PDMS blocker assay. The migration was recorded 30 minutes after the removal of the PDMS blocker. An image was taken every 4 minutes. The duration of the video is 2 hours. The corresponding PIV analysis was performed as described in the material and method section.

**Video 2: Endothelial collective migration on a converging pattern**

HUVEC were seeded on a plasma lithography defined converging pattern created on a 60 mm petri dish. Cell migration was induced by the PDMS blocker assay. The migration was recorded 30 minutes after the removal of the PDMS blocker. An image was taken every 3 minutes. To reduce the size of the video, the time span between two images is 9 minutes. The duration of the video is 17 hours.

**Video 3: Endothelial collective migration on a diverging pattern**

HUVEC were seeded on a plasma lithography defined diverging pattern created on a 60 mm petri dish. Cell migration was induced by the PDMS blocker assay. The migration was recorded 30 minutes after the removal of the PDMS blocker. An image was taken every 3 minutes. To reduce the size of the video, the time span between two images is 6 minutes. The duration of the video is 12 hours.

**Video 4: Endothelial collective migration of a completing cell pattern**

HUVEC were seeded on a plasma lithography defined trapezoidal pattern created on a 60 mm petri dish. Two PDMS blockers were placed in the two ends. The migration of the completing cell pattern was recorded 30 minutes after the removal of the PDMS blockers. An image was taken every 6 minutes. The duration of the video is 7.5 hours.
